# Spatial and time-resolved properties of emission enhancement in polar/semi-polar InGaN/GaN by surface plasmon resonance

**DOI:** 10.1515/nanoph-2023-0758

**Published:** 2024-02-07

**Authors:** Kento Ikeda, Kanata Kawai, Jun Kametani, Tetsuya Matsuyama, Kenji Wada, Narihito Okada, Kazuyuki Tadatomo, Koichi Okamoto

**Affiliations:** Department of Physics and Electronics, Osaka Metropolitan University, Gakuen-cho, Naka-ku, Sakai-shi, Osaka 599-8531, Japan; Department of Electrical and Electronic Engineering, Yamaguchi University, Tokiwadai, Ube-shi, Yamaguchi 755-8611, Japan

**Keywords:** surface plasmon resonance, plasmonics, InGaN/GaN, semi-polar, light-emitting diodes, micro-photoluminescence

## Abstract

Light-emitting diodes (LEDs) are widely used as next-generation light sources because of their various advantages. However, their luminous efficiency is remarkably low at the green-emission wavelength. The luminous efficiencies of InGaN/GaN quantum wells (QWs) significantly decrease with increasing indium content in the green wavelength region, mainly owing to the quantum-confined Stark effect (QCSE). This green gap problem can be solved using QWs grown on semi-polar GaN substrates, such as the {11–22} planes, to reduce the QCSE. We propose that the use of surface plasmons (SPs) is a promising way to improve the light emission efficiency of light-emitting materials such as InGaN/GaN QWs. SP resonance increases the spontaneous emission rates of the excited states, causes a relative reduction in non-radiative relaxation, and ultimately increases the internal quantum efficiencies. In this study, the light emissions of InGaN/GaN QWs grown on polar and semi-polar GaN were investigated using micro-photoluminescence (PL). We successfully enhanced the light emission of semi-polar GaN via SP resonance. The PL peak intensities and wavelengths were mapped and compared to determine the underlying mechanisms. We also measured the emission lifetimes by time-resolved PL and interpreted the detailed mechanism of SP-enhanced emissions. It was found that SP resonances can control not only the emission efficiency but also the exciton dynamics, such as exciton localization effects, QCSE screening, and defect level saturation. We conclude that the green gap problem can be solved by SP-enhanced light emission in semipolar InGaN/GaN.

## 1 Introduction

Light-emitting diodes (LEDs) are widely used as next-generation light sources owing to their various advantages such as high emission efficiency, wavelength selectivity, small size, light weight, and long service life, compared with conventional fluorescent lamps. In recent years, various studies have been conducted to improve the light-emission efficiency of LEDs. Technically, the crystal quality has been improved to suppress the phonon conversion of excitons and improve the internal quantum efficiency [1]. In addition, light extraction efficiency has been improved by microfabrication on the substrate [2]. However, the light emission efficiency of red and blue among the three primary colors of light is approximately 50 % and 80 %, respectively; it is approximately 30 % for green, and that in the ultraviolet region is low. This is known as the green gap problem, in which light emission efficiencies drop significantly at green emission wavelengths [3], [4]. In InGaN/GaN semiconductor quantum wells (QWs) used in light-emitting devices, the low light emission efficiency at green wavelengths is thought to be caused by the inhomogeneity of the indium composition in the crystal of the QW layer. In green LEDs, the high indium composition of the InGaN layer causes distortions in the crystal owing to lattice mismatch. This causes an internal electric field, which in turn causes the quantum-confined Stark effect (QCSE), leading to a decrease in the carrier coupling efficiency, and thus a decrease in the light emission efficiency [5], [6], [7], [8].

A solution to the green gap problem is a method to reduce the internal electric field by fabricating LED devices on a semi-polar plane such as the {11–22} plane, which is different from the commonly used {0001} plane (c-plane) [9]. The method is to grow a flat semi-polar GaN layer on patterned sapphire substrates (PSS) [10].

Another reason for the reduction in emission efficiencies with increasing indium composition in the green wavelength region is related to exciton dynamics and localization processes depending on the nanostructure. To understand the important factors that determine the optical properties of InGaN QWs, we investigated the time- and spatial-resolved direct observations of diffusion processes and radiative and non-radiative recombination processes of excitons by using third-order nonlinear spectroscopy [11], scanning near-field microscopy [12] and a new method developed by combining the previous two techniques [13]. We found that the special fluctuation of indium composition in the InGaN layer leads to the localization centers of excitons and, accordingly, the emission efficiency becomes high. However, the localization of excitons becomes weaker with increasing indium composition due to phase separation in the crystal [14], [15].

Based on these results, we propose that controlled spatial inhomogeneity in the InGaN active layer is more effective than increasing the crystal quality to improve the emission efficiency of InGaN LEDs in the green light region [16]. To demonstrate, we fabricated arrayed nanopillar structures of InGaN using electron beam lithography and a dry etching process and achieved bright green emissions [17]. We propose that the use of surface plasmons (SPs), which are plasma oscillations of free electrons at the metal-dielectric interface, is a promising way to improve the light emission efficiency of light-emitting materials. In 2004, we presented the first report of large photoluminescence (PL) enhancements for InGaN/GaN QW coated with Ag thin films [18]. Subsequently, many high-efficiency plasmonic devices have been attempted to be developed in recent years [19], [20], [21], [22], [23].

In this study, to improve the light emission efficiency of blue/green-emitting InGaN/GaN, we attempted to enhance the light emission by combining semi-polar planes and SP resonance, and investigated the luminescence mechanism. Therefore, we performed spatial- and time-resolved PL measurements. Although there have been many reports on SP-enhanced luminescence using InGaN/GaN QWs, the detailed enhancement mechanism related to exciton emission is still unclear. The internal quantum efficiency of emission from InGaN/GaN QWs strongly depends on the exciton dynamics in the active layer, such as exciton localization [24], [25] and QCSE [26], [27]. The spatially resolved evaluation of PL features by micro-PL measurements is effective in measuring and explaining exciton dynamics. Recently, we reported that special inhomogeneities of both the PL peak intensities and wavelengths were observed by the micro-PL measurements of polar InGaN/GaN QWs, and the features were changed by the SP resonance [28], [29], [30]. In this study, we investigated the micro-PL with the SP resonance for semi-polar InGaN/GaN QWs and compared the results with those of polar samples to investigate whether SP-enhanced emission is still effective for semi-polar InGaN/GaN. We also measured the emission lifetime using time-resolved PL measurements to interpret the detailed mechanism of the SP emission enhancement.

## 2 Methods

### 2.1 Samples

**Figure 1:**
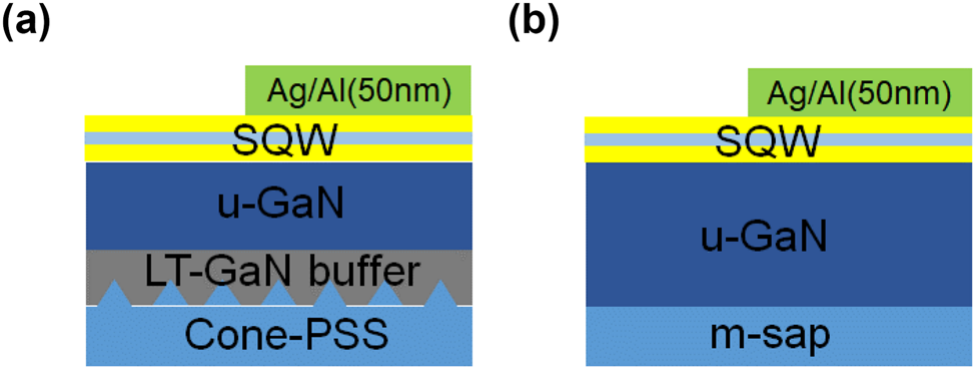
Schematic of polar (a) and semi-polar (b) InGaN/GaN QW sample structure.

Polar and Semi-polar InGaN/GaN single QW were grown via metal-organic vapor phase epitaxy (MOVPE). Figure 1 shows schematics of the polar (a) and semi-polar (b) sample structures. The thickness of the InGaN/GaN layer is 2.5 nm/12.5 nm for the polar sample and 3.5 nm/14.2 nm for the semipolar sample; the thickness of the undoped GaN layer is 2.5 μm for both. The polar {0001} sample was grown on a cone-shaped patterned c-plane sapphire substrate, which is commonly used to improve the light extraction efficiency [31]. The semi-polar {11–22} sample was grown on an m-plane sapphire substrate [32]. A single InGaN QW layer with 3 nm thickness was grown on a GaN layer on a sapphire substrate. Because the QW layer cannot be excited from the front by a laser when metal films are deposited onto the samples, the back of the sapphire substrate is polished to create a mirror-finished flat surface that suppresses light scattering to efficiently excite and detect the QW from the back. Four types of samples were used: polar/semi-polar, blue-emitting, and green-emitting. The In composition of the InGaN well layer is 10 % and 25 % for blue and green emission, respectively.

Half of the sample surface was covered with heat-resistant tape. After the pressure in the chamber was reduced to less than 2.0 × 10^−3^ Pa, Ag or Al with a thickness of 50 nm was deposited by high-vacuum thermal evaporation (Sanyu electron SVC-700TM). A deposition rate of 1.2–2.0 Å*s* ^−1^ was adopted to create a metal film with a grain size that is expected to increase the light emission efficiency. Atomic force microscopy (AFM) images of the metal films are shown in Figure S1. All of them have a grain structure of 50–100 nm, which helps to extract light from the surface plasmon [33].

### 2.2 PL mapping

PL spectra were measured using a fluorescence microscope (Olympus BX51TRF) with a mercury lamp for excitation. The detailed setup was published in our previous reports [28], [29], [30]. The incident wavelengths were filtered by one of the two band-pass filters, either 390–400 nm or 400–410 nm, for the blue- and green-emitting samples. The moving stage of the fluorescence microscope was electrically controlled using a stepper motor, with a minimum step width of 4 nm. The size of the scanned area was 200 μm × 200 μm, and PL spectra were obtained for each pixel (4 μm × 4 μm) using a spectrophotometer (Spectra Pro 2300i) and a CCD camera (Roper Scientific, PIXIS 100B-3). All the PL spectra obtained at each pixel were fitted with a Gaussian function in the wavelength regions near the PL peaks (Figure S2). More accurate values of the PL peak wavelength and intensity, excluding interference and noise, were obtained from the Gaussian fitting result [28], [29], [30].

### 2.3 Time-resolved PL measurement

An InGaN pulsed laser (Hamamatsu Photonics PLP-10) was used for excitation, and time-resolved PL was detected using a photomultiplier tube and time-correlated single-photon counter (PicoQuant PicoHarp 300). The light emission from the sample caused by laser excitation was focused by two plano-convex lenses. To eliminate the excitation light, a long-pass filter that transmits only light beyond 420 nm was incorporated into the optical system, and the measurement was performed in an environment where external light was blocked as much as possible.

## 3 Results and discussion

### 3.1 Blue-emitting samples

**Figure 2:**
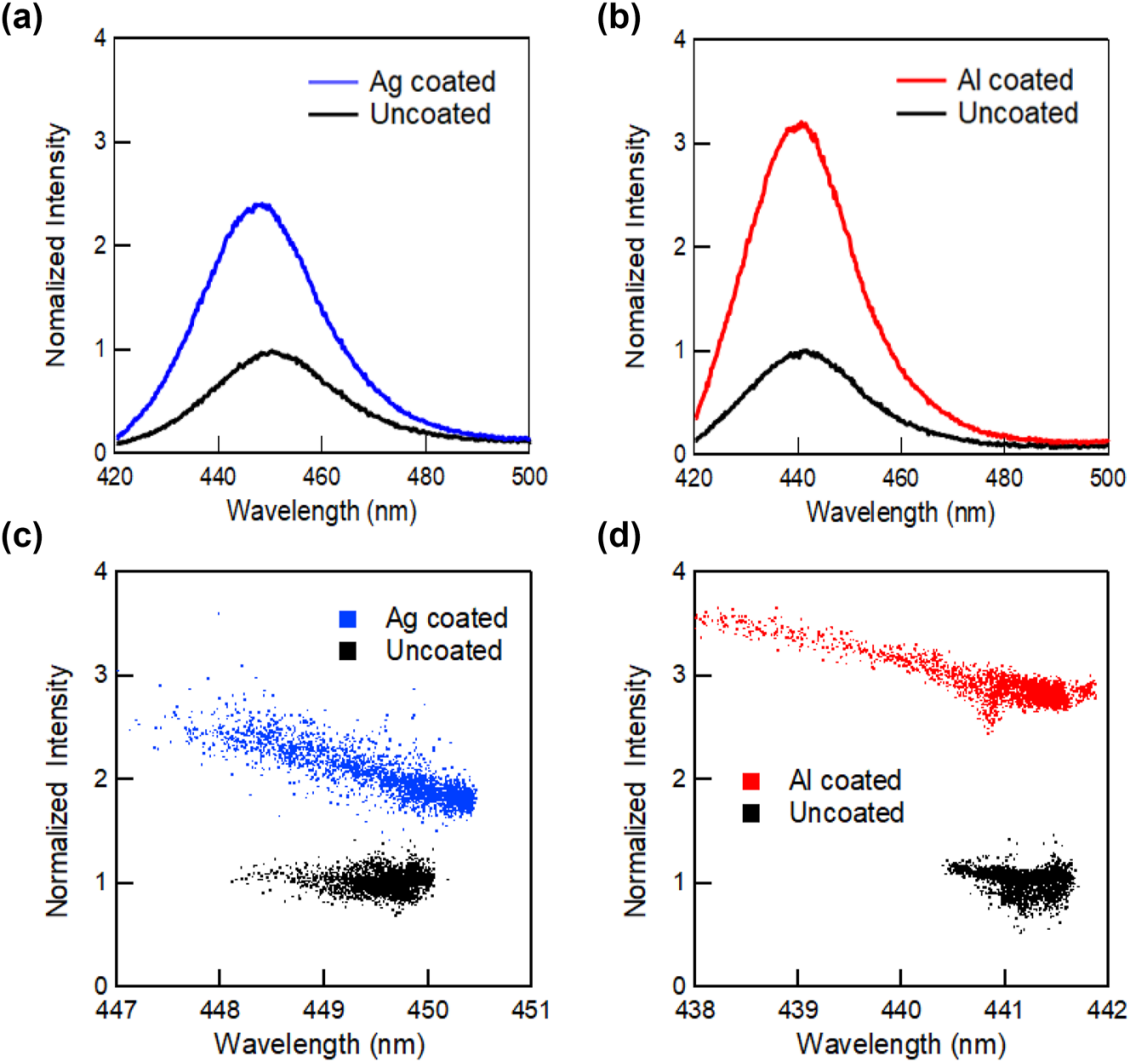
PL spectra for polar blue-emitting InGaN/GaN QW with Ag (a) and Al (b) coating, and the correlations between the PL peak intensity and wavelength for the ample with Ag (c) and Al (d). The PL peak intensities were normalized by using the mean of the PL peak intensities of the uncoated samples.

**Figure 3:**
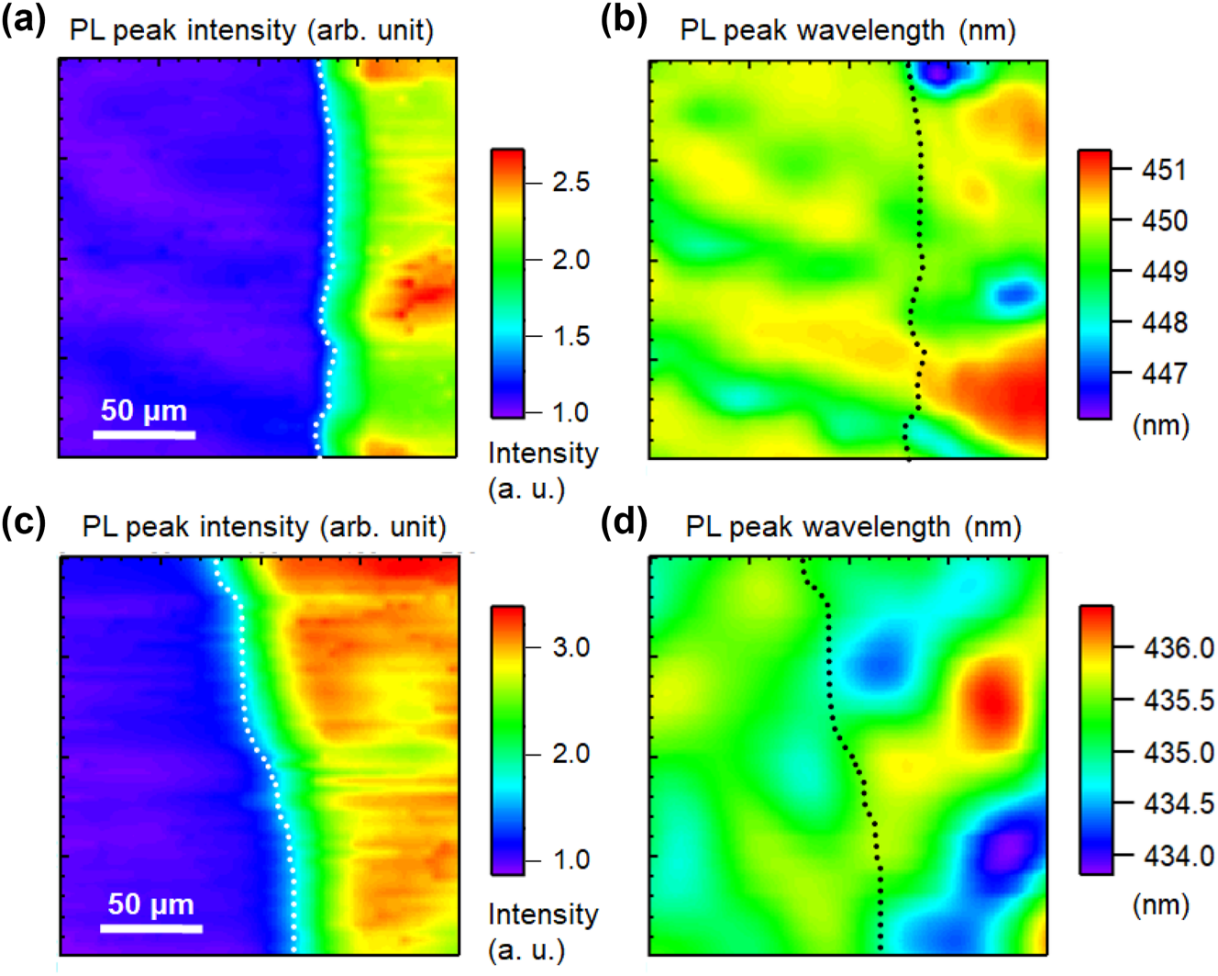
PL peak intensity (a) and wavelength (b) mapping for the polar blue-emitting InGaN/GaN with (right side) and without (left side) Ag-coated regions. PL peak intensity (c) and wavelength (d) mapping for the polar blue-emitting InGaN/GaN with (right side) and without (left side) Al-coated regions. The dotted lines show the boundary of the coated metal layers.

Figure 2 shows the PL spectra of polar blue-emitting InGaN/GaN QW samples with Ag (a) and Al (b) coatings, which were measured near the boundary between the metal-coated and uncoated regions to avoid the influence of the wavelength fluctuation of the sample. The PL peak intensities of the uncoated samples were normalized to 1. For both samples, no change was observed in the wavelength for either the Ag- or Al-coated regions. Figure 3 shows the PL peak intensity (a) and wavelength (b) mapping for polar blue-emitting InGaN/GaN with (right side) and without (left side) Ag-coated regions, and a similar PL peak intensity (c) and wavelength (d) mapping with and without Al-coated regions. Dotted lines indicate the boundaries of the coated metal layers. All the obtained mappings show micrometer-scale spatial inhomogeneities owing to the distributions of indium compositions. Emission enhancement and wavelength changes caused by SP resonance were observed in micro-PL mapping. The overall PL peak intensities in the Ag-coated region were enhanced by approximately 2-fold, whereas those in the Al-coated region were enhanced by approximately 3-fold compared with those in the uncoated regions. Figure 2(c) and (d) suggest a correlation between the PL peak wavelength and intensity, indicating that there is a negative correlation (PL intensities were weaker at longer wavelengths) in the Ag-coated and Al-coated regions. The PL peak intensities were normalized to the mean of the PL peak intensities of the uncoated samples. The PL peak wavelength plots were spread over a wider wavelength range in the coated region for both the metals.

**Figure 4:**
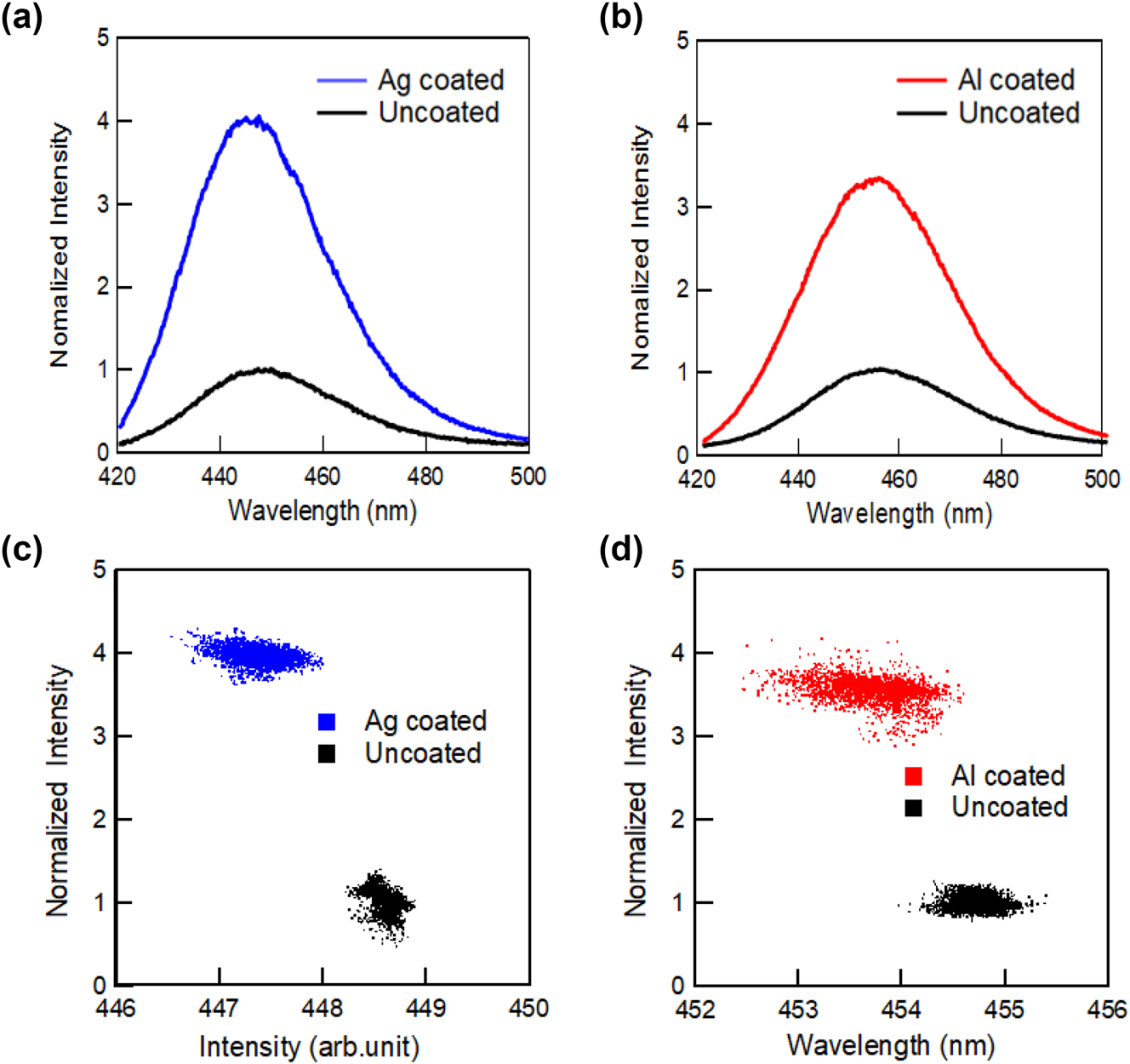
PL spectra for semi-polar blue-emitting InGaN/GaN QW with Ag (a) and Al (b) coating, and the correlations between the PL peak intensity and wavelength for the ample with Ag (c) and Al (d). The PL peak intensities were normalized by using the mean of the PL peak intensities of the uncoated samples.

**Figure 5:**
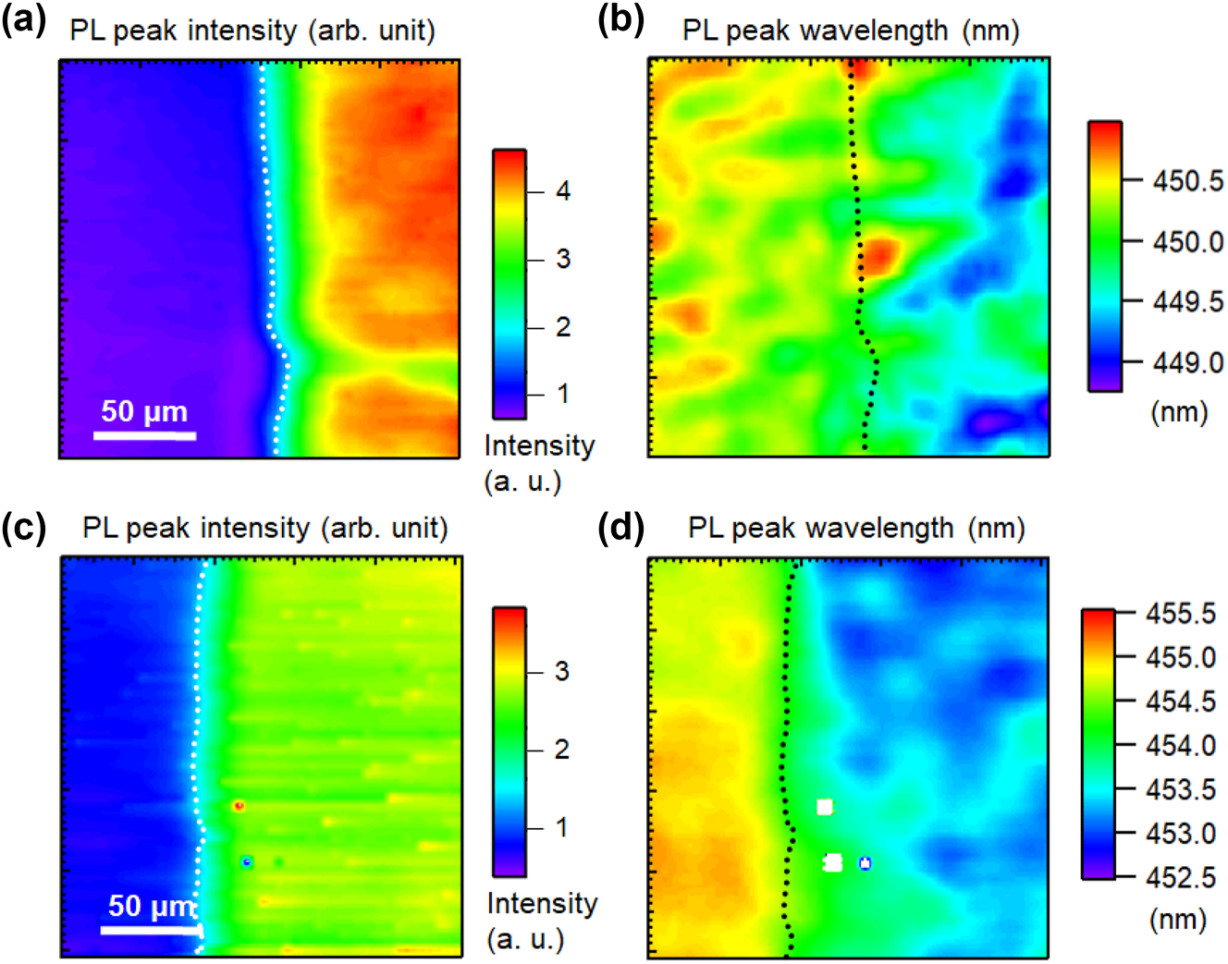
PL peak intensity (a) and wavelength (b) mapping for the semi-polar blue-emitting InGaN/GaN with (right side) and without (left side) Ag-coated regions. PL peak intensity (c) and wavelength (d) mapping for the semi-polar blue-emitting InGaN/GaN with (right side) and without (left side) Al-coated regions. The dotted lines show the boundary of the coated metal layers.

Figure 4 shows the PL spectra of semi-polar blue-emitting InGaN/GaN QW samples with Ag (a) and Al (b) coatings and the correlations between the PL peak intensity and wavelength for the samples with Ag (c) and Al (d). Figure 5 shows the PL peak intensity (a) and wavelength (b) mapping for the semi-polar blue-emitting InGaN/GaN with (right side) and without (left side) Ag-coated regions and a similar PL peak intensity (c) and wavelength (d) mapping with and without Al-coated regions. Similar to the polar sample, the emission was strongly enhanced by the SP resonance, while the wavelength distributions became significantly wider. Figure 4(c) and (d) show that the correlation between the PL peak wavelength and intensity is also different from that of the polar sample. The overall PL peak intensities in the Ag-coated region were enhanced by approximately 4-fold, whereas those in the Al-coated region were enhanced by approximately 3.5-fold compared to those in the uncoated regions. For the semi-polar sample, the distributions were not broad, and the PL wavelengths were blue-shifted by metal coating. This may be because QCSE was reduced in the semi-polar sample, and the exciton localization effect became dominant in the luminescence mechanism.

The observed PL enhancements may include contributions from the mirror effect, because the deposited metal films also act as mirrors. However, the mirror effect should not be the dominant factor in the PL enhancement for the following reasons. First, the enhancement factors were dramatically reduced with increasing distance between the QWs and the metal interface [16], [18]. Second, the internal quantum efficiencies estimated by the temperature dependence of the PL intensities increased. Third, the time-resolved PL measurements revealed that the PL lifetimes were shortened by SP resonance [16], [18]. These phenomena cannot be explained by the mirror effect alone, and the resonance mechanism between excitons and SPs must be considered [34], [35]. The SP resonance energies (wavelengths) are estimated as 5.7 eV (220 nm), 2.9 eV (430 nm), and 2.3 eV (540 nm) for Al/GaN, Ag/GaN, and Au/GaN interfaces, respectively, from their dielectric functions and surface plasmon dispersion relationships [18]. This suggests that Al, Ag, and Au should be suitable for use in the UV, blue, and red regions of the spectrum, respectively.

**Figure 6:**
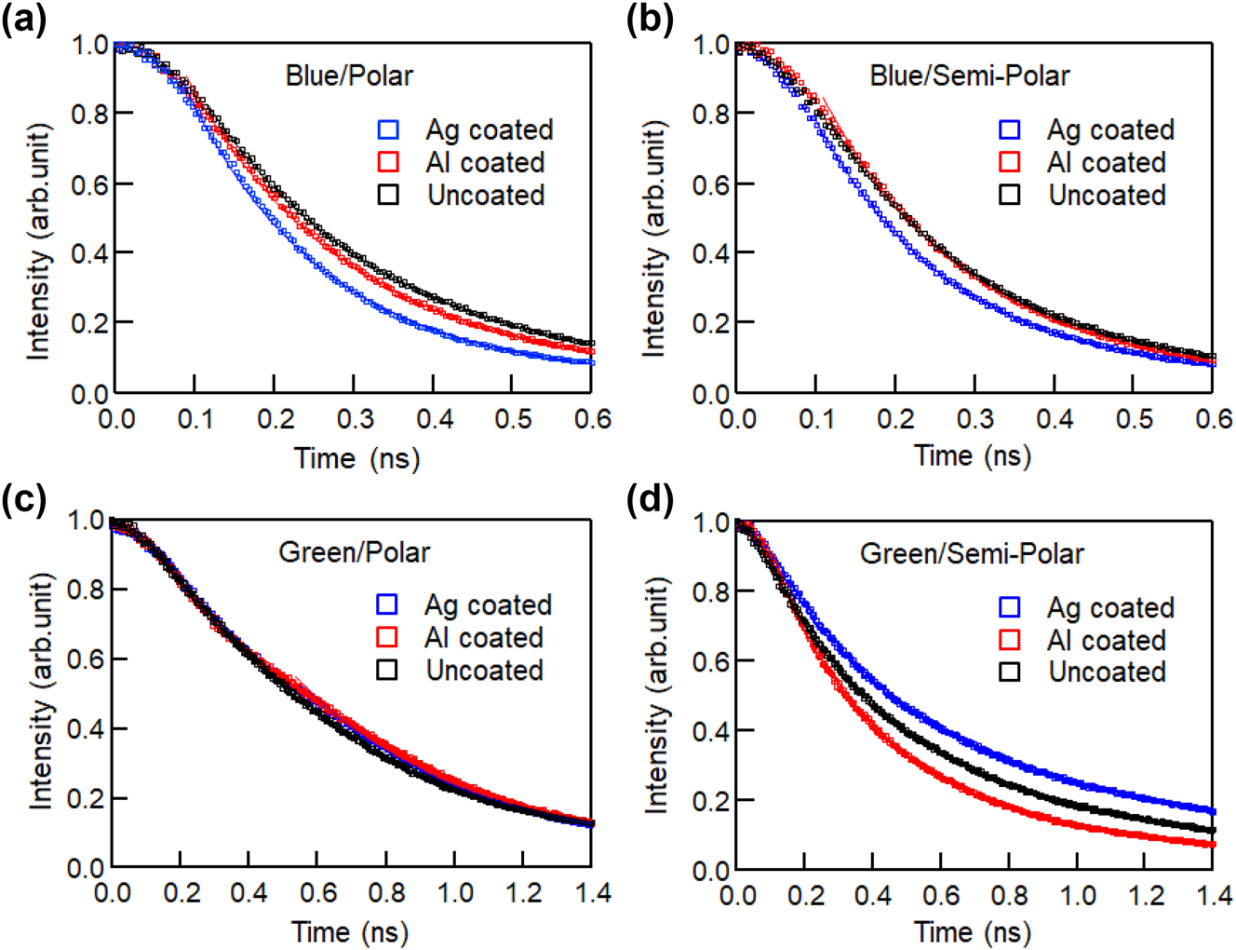
PL time profiles of polar (a) and semi-polar (b) sample with blue emission and polar (c) and semi-polar (d) sample with green emission of InGaN/GaN QW.

Time-resolved PL measurements were performed to confirm the contribution of SP resonances to the obtained PL enhancements. Figure 6 shows the PL time profiles of the polar (a) and semi-polar (b) samples with blue emission from the InGaN/GaN QW. The PL lifetime of the blue-emitting sample was shortened by metal coating on both the polar and semi-polar samples. The PL time profiles were fitted with a double exponential decay function to obtain the PL lifetimes. PL decay curves usually exhibit a double exponential decay model that includes fast and slow decay. These two decays correspond to transitions to strongly localized states and exciton recombination. When performing a mechanism analysis, it is necessary to analyze the fast and slow decay in TRPL, respectively, to verify the mechanism proposed by the authors through a detailed analysis. However, in the present sample, the fast-decay component is dominant and the slow component is difficult to analyze with high accuracy. Therefore, in this time, we determined the PL lifetimes of the fast-decay components of the dominant components. In the polar samples, the PL lifetimes of the metal-uncoated, Ag-coated, and Al-coated regions were 0.21 ns, 0.17 ns, and 0.20 ns, respectively. In the semi-polar samples, the PL lifetimes of the metal-uncoated, Ag-coated, and Al-coated regions were 0.19 ns, 0.17 ns, and 0.18 ns, respectively. These results suggest that SP resonance is a type of Purcell effect and that an increase in emission rates contributes to the improvement of light emission efficiencies. We explained the emission enhancement due to the Purcell effect as follows: the radiation from the exciton resonates with the electric field oscillations of the surface plasmon, acting as a photo-antenna. This resonance increases the spontaneous emission rate and suppresses the non-radiative process, thereby enhancing the internal quantum efficiency (IQE). Even if the exciton is trapped in a deeper level, if the energy level of the exciton matches the energy level of the plasmon, this effect will promote luminescence. This was confirmed by microscopic PL [28], [29], [30]. The PL lifetime of the semi-polar sample was shorter, even in the metal-uncoated region, owing to the reduction in QCSE. We found that the SP resonance further shortened the PL lifetime, even for semi-polar samples with short PL lifetimes.

Herein, we explain the luminescence mechanism based on the obtained experimental results. Ag and Al exhibit different SP resonance properties. The resonance wavelength of the SP-propagating mode at the Ag/GaN interface is approximately 440 nm, which is close to the blue emission. Thus, the blue emissions from the samples can couple with the SP at the Ag/GaN interface. SP coupling increased the emission rate of the blue emission, as seen in the shortened PL lifetime in Figure 6(a). However, the resonance wavelength of the SP-propagating mode at the Al/GaN interface was approximately 250 nm, and the SP resonated in the longer-wavelength region. Therefore, the SP can couple with not only the blue emission, but also the excitation light (400 nm) at the Al/GaN interface, which also increases the exciton density. The PL lifetime was also shortened by the Al coating, as shown in Figure 6(a), suggesting that the SP-enhanced emission mechanism with the Al coating may be similar to that with the Ag coating.

The correlation plots between the PL peak intensities and wavelengths shown in Figure 2(c) and (d) suggest that the distribution of the PL peak wavelength becomes broad for both Ag and Al coatings, whereas the linewidth of the spectra shown in Figure 2(a) and (b) remains unchanged. It is known that QCSE can be screened when the exciton densities are high after photoexcitation [36], [37]. The screening of QCSE causes a blue shift in the PL intensities. After excitation, the screening effect was reduced with decreasing exciton density, and the QCSE became more remarkable. This reduction could cause a red shift in the PL intensity. Because the QCSE brings about a PL peak shift in this way, the observed broad distribution of PL wavelengths in polar InGaN/GaN may be attributed to the spatial variation of the QCSE. The SP-enhanced emission rate could reduce the screening effect of QCSE and make QCSE more remarkable. However, the Al coating increases the exciton density, which promotes QCSE screening (reduces QCSE), resulting in a blueshift. This could be the reason for the much broader distribution of the PL peak wavelength with the SP resonance with the metal coating. A schematic view of the mechanism of PL enhancement by the enhancement and reduction of QCSE with SP resonance is shown in Figure S3.

In contrast, in the semi-polar sample, the QCSE was reduced and the effect of exciton localization became more pronounced. The potential energy level of the InGaN layer fluctuates because of the inhomogeneity of the indium composition, and the generated excitons are localized at a lower potential level, which is the exciton localization effect. When this sample is coated with Ag or Al, the exciton can couple to the SP and the emission rate can be increased. If the SP resonance increases the emission rate faster than the exciton localization, the exciton localization effects can be canceled, which causes a blueshift of the PL peak wavelength, as shown in Figure 4(c). Therefore, the distribution of the PL peak wavelength was not broadened compared to that of the polar sample and was blue-shifted for both metal coatings. In the case of the Al coating, the exciton density increased owing to the resonance of the excitation light and SP, and the localized levels were filled, resulting in emission from higher levels than those in the uncoated case. This also causes a blue shift in the PL spectra. A schematic view of the PL enhancement mechanism by cancelation and saturation of the exciton localization effect with SP resonance is shown in Figure S4. For the polar sample, the QCSE effect is dominant, but the exciton localization effect may also be present simultaneously. For this reason, Figure 2(c) shows both blueshift and redshift depending on the location.

The QCSE effect was reduced for the semi-polar sample, but the emission was still enhanced by the SP resonance. This suggests that the cancelation of the QCSE for the semi-polar sample and the emission enhancement effect by the SP resonance do not cancel each other out, but coexist instead. In the semi-polar sample, the lifetime in the metal-uncoated region is shorter than that in the polar sample. In addition, metal coating shortened the lifetime. From these results, we concluded that the PL enhancement effect by SP resonance should be sufficient even for semi-polar samples where the QCSE effect is reduced.

### 3.2 Green-emitting samples

**Figure 7:**
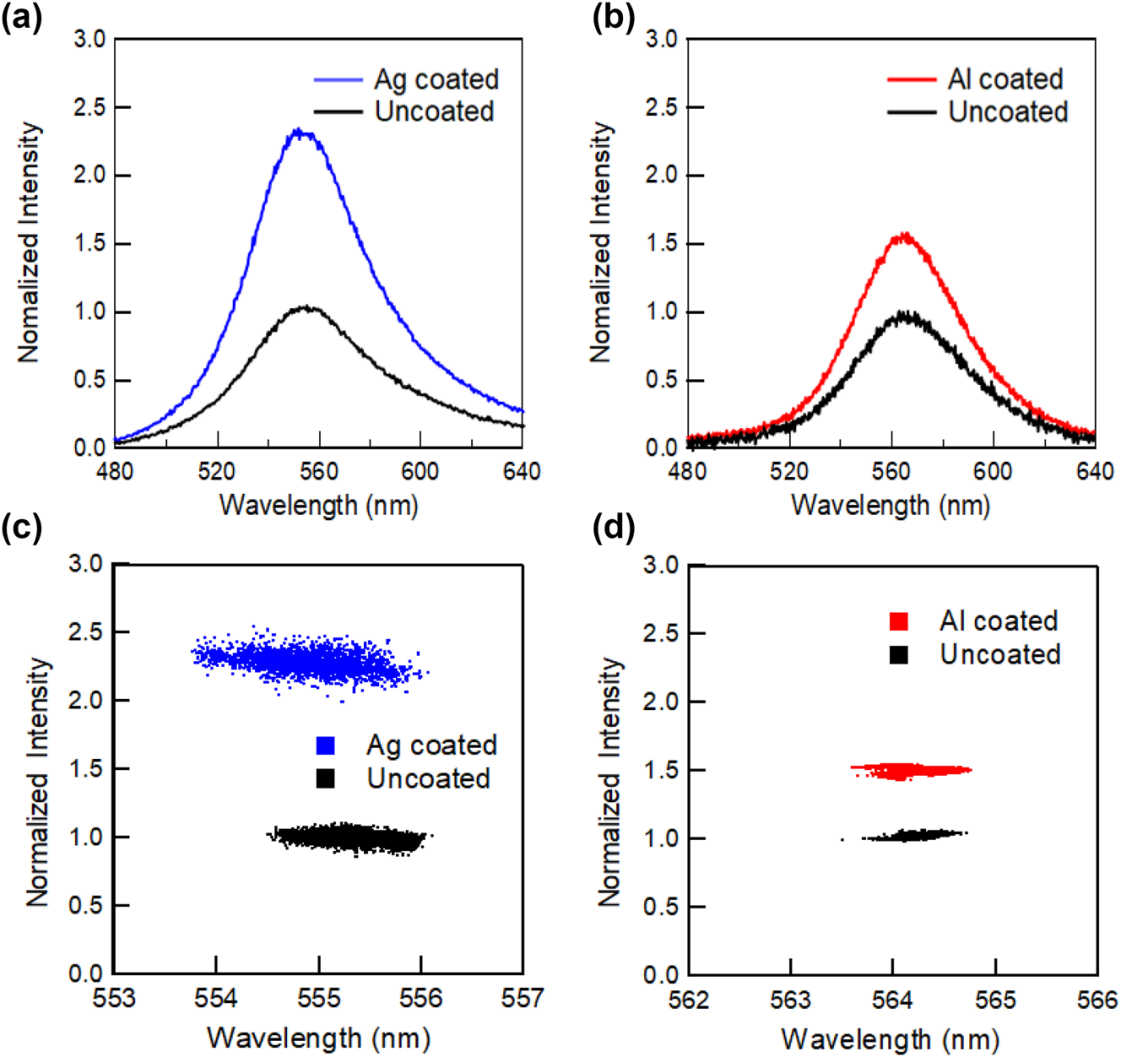
PL spectra for polar green-emitting InGaN/GaN QW with Ag (a) and Al (b) coating, and the correlations between the PL peak intensity and wavelength for the ample with Ag (c) and Al (d). The PL peak intensities were normalized by using the mean of the PL peak intensities of the uncoated samples.

**Figure 8:**
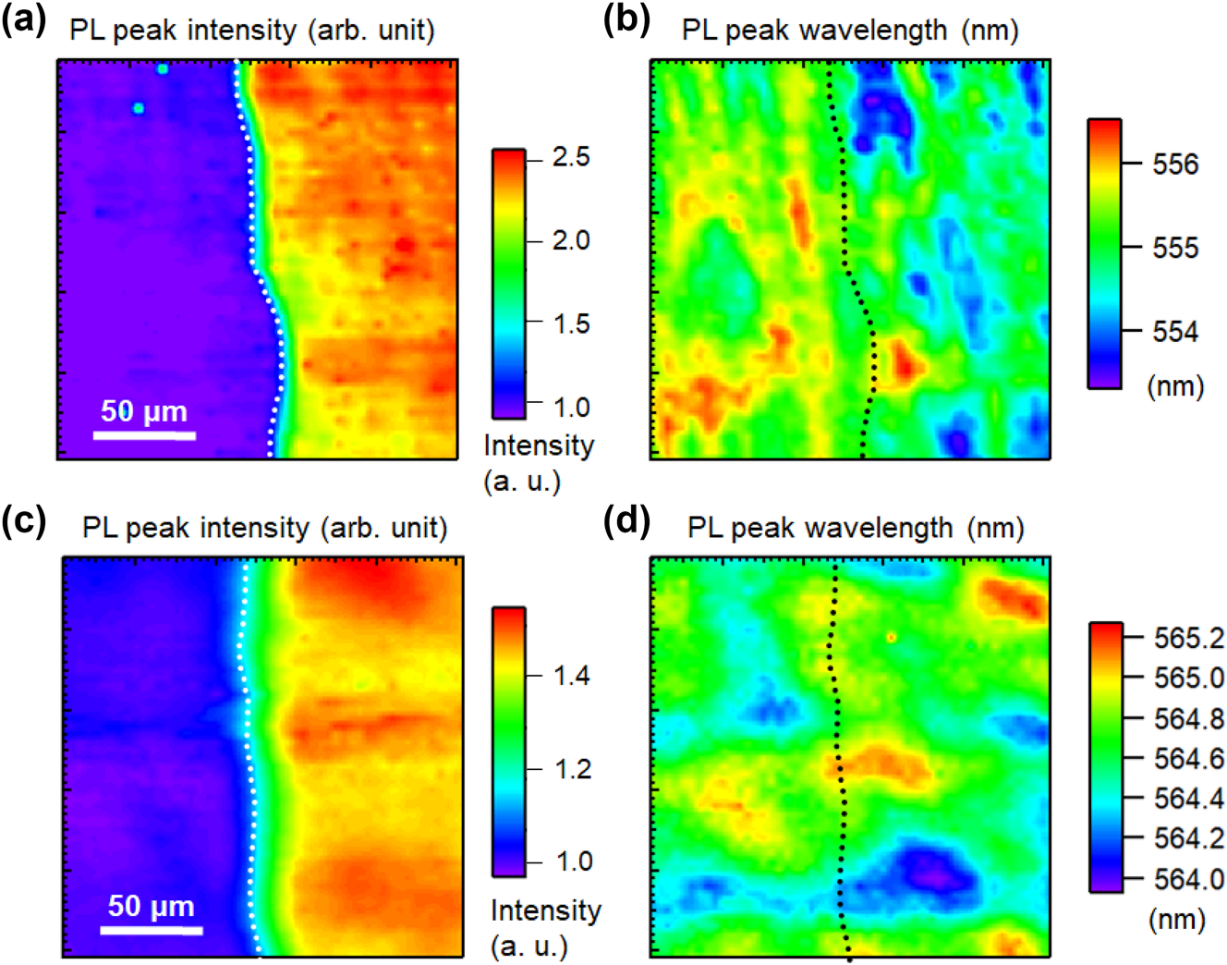
PL peak intensity (a) and wavelength (b) mapping for the polar green-emitting InGaN/GaN with (right side) and without (left side) Ag-coated regions. PL peak intensity (c) and wavelength (d) mapping for the polar green-emitting InGaN/GaN with (right side) and without (left side) Al-coated regions. The dotted lines show the boundary of the coated metal layers.

We performed a similar investigation of InGaN/GaN with green emission. Figure 7 shows the PL spectra of polar (a) and semi-polar (b) green-emitting InGaN/GaN QWs with Ag and Al coatings and the correlations between the PL peak intensity and wavelength for the samples with Ag (c) and Al (d). The spectra of the polar sample were almost unaffected by the Ag and Al coatings, whereas those of the semi-polar sample were significantly red-shifted by the Ag coating and significantly blue-shifted by the Al coating. The properties of green emissions were found to differ from those of blue emissions. Figure 8 shows the PL peak intensity (a) and wavelength (b) mapping for the polar green-emitting InGaN/GaN with and without Ag-coated regions and a similar PL peak intensity (c) and wavelength (d) mapping with and without Al-coated regions. We observed emission enhancement and wavelength shift caused by SP resonance by micro-PL mapping, similar to blue-emitting samples. The overall PL peak intensities in the Ag-coated region were enhanced by approximately 2.5-fold, whereas those in the Al-coated region were enhanced by approximately 1.5-fold compared to those in the uncoated regions. For the green-emitting sample, the luminescence mechanism may be more affected by QCSE owing to its large indium content, but the degree of influence may vary depending on the location. In addition, the defect density may also be high because of the high indium content.

Figure 7(c) and (d) show that there is no correlation between the PL peak wavelength and intensity. For both metal-coated regions, the PL peak positions and distributions were almost the same, while the peak intensities were enhanced. For the blue-emitting sample, the effect of QCSE was screened by photoexcitation, whereas for the green-emitting sample, QCSE was not sufficiently screened because the QCSE effect was much stronger. Therefore, the QCSE was not affected by the SP resonance and the PL peak shift was not observed with the metal coating for the green-emitting InGaN/GaN sample. Figure 6(c) shows the PL time profiles of the polar sample with green emission of the InGaN/GaN QW. The PL lifetimes of the metal-uncoated, Ag-coated, and Al-coated regions were 0.48 ns, 0.46 ns, and 0.47 ns, respectively. The PL time profiles did not change significantly for either of the metal coatings. This suggests that the screening of QCSE caused by SP resonance should be smaller than that of the blue-emitting samples.

**Figure 9:**
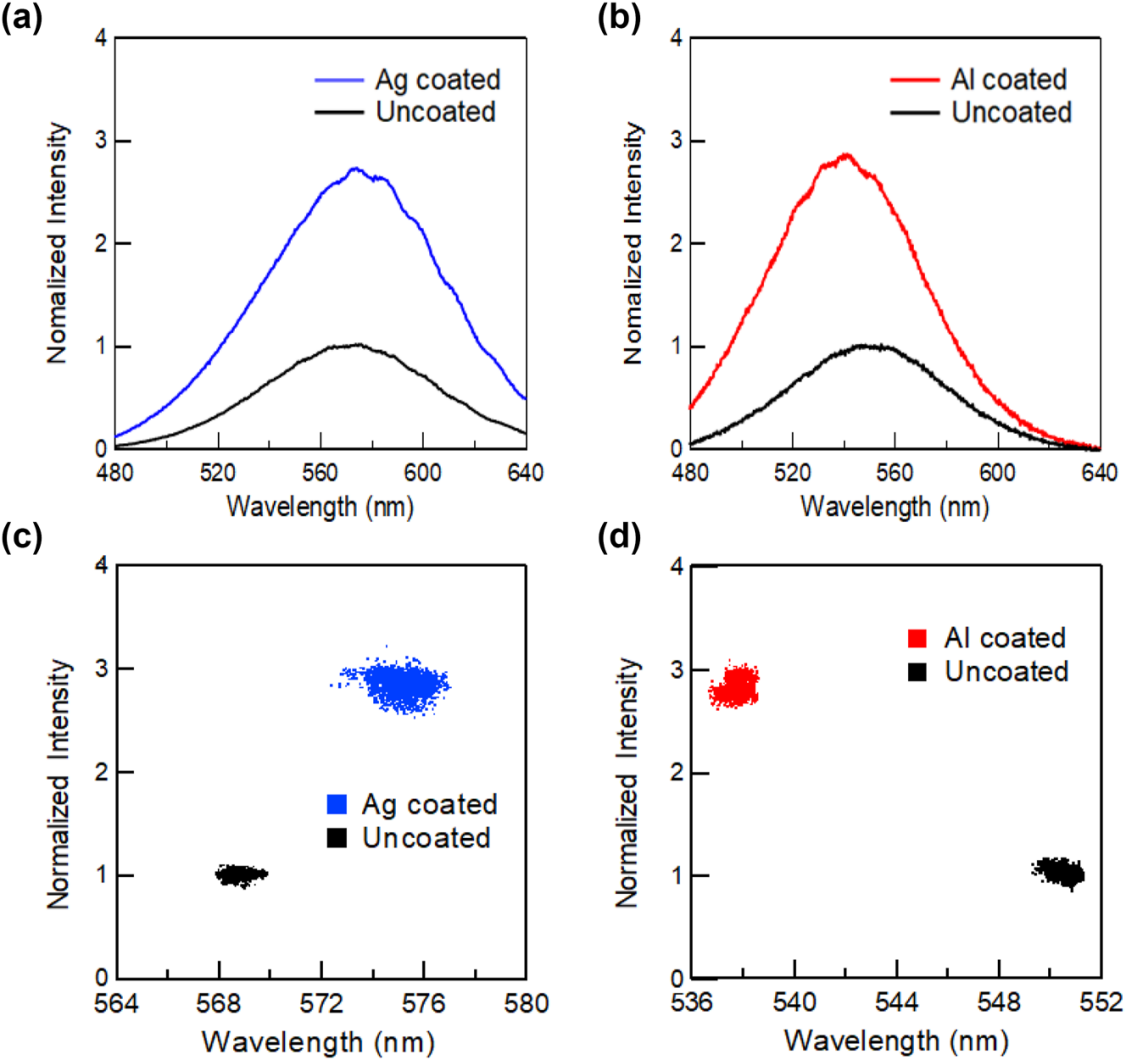
PL spectra for semi-polar green-emitting InGaN/GaN QW with Ag (a) and Al (b) coating, and the correlations between the PL peak intensity and wavelength for the ample with Ag (c) and Al (d). The PL peak intensities were normalized by using the mean of the PL peak intensities of the uncoated samples.

**Figure 10:**
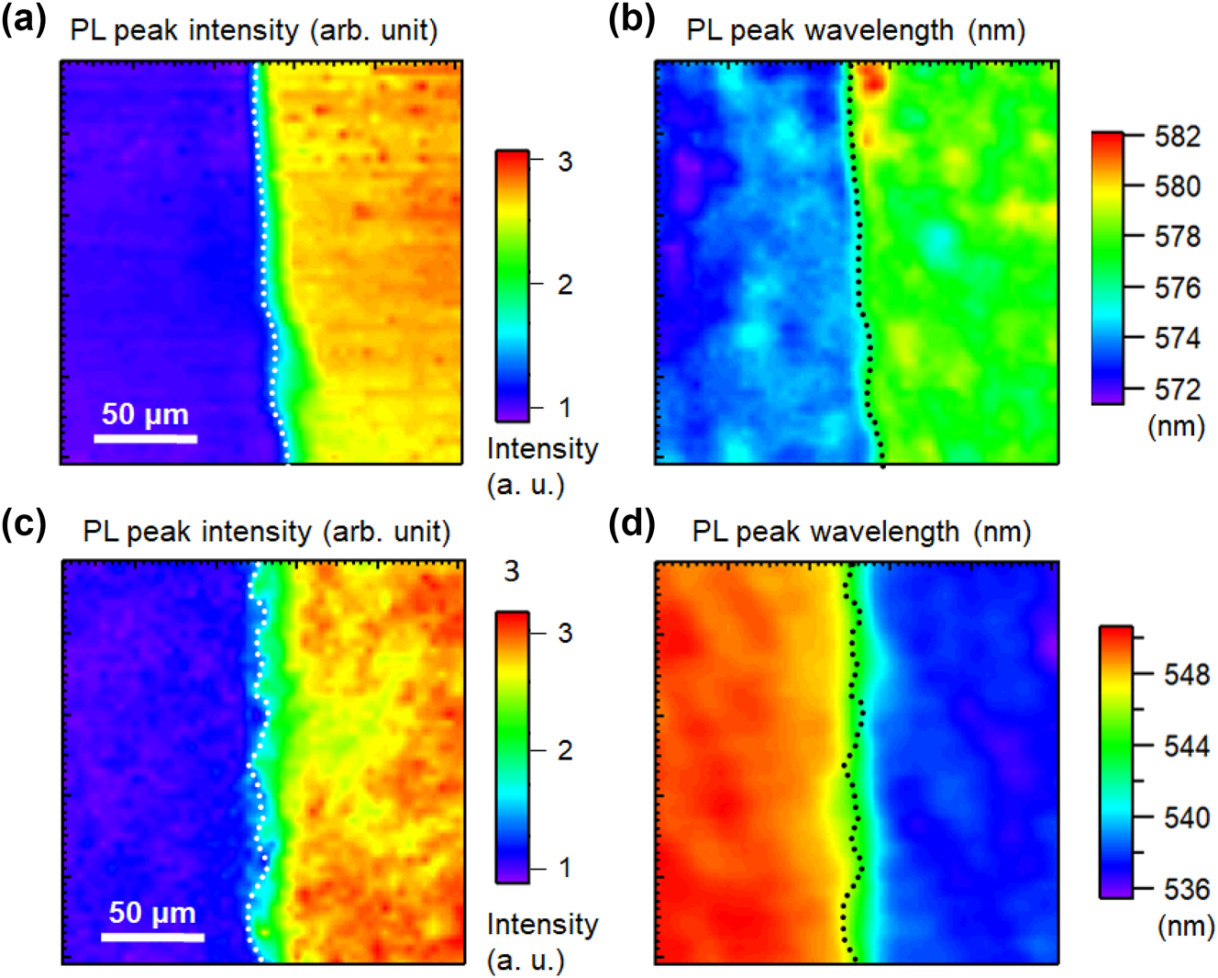
PL peak intensity (a) and wavelength (b) mapping for the semi-polar green-emitting InGaN/GaN with (right side) and without (left side) Ag-coated regions. PL peak intensity (c) and wavelength (d) mapping for the semi-polar green-emitting InGaN/GaN with (right side) and without (left side) Al-coated regions. The dotted lines show the boundary of the coated metal layers.

Figure 9 shows the PL spectra of the semi-polar green-emitting InGaN/GaN QW samples with Ag (a) and Al (b) coatings, and the correlations between the PL peak intensity and wavelength for the samples with Ag (c) and Al (d). Figure 10 shows the PL peak intensity (a) and wavelength (b) mapping for the semi-polar green-emitting InGaN/GaN with and without Ag-coated regions and a similar PL peak intensity (c) and wavelength (d) mapping with and without Al-coated regions. The overall PL peak intensities in the Ag- and Al-coated regions were approximately 3-fold higher than those in the uncoated regions. In the PL spectra, the PL peak wavelength of the semi-polar sample was significantly red-shifted by the Ag coating and significantly blue-shifted by the Al coating. The spectral distribution of the SP peak wavelengths was narrow both with and without metal coating because the QCSE was canceled for the semi-polar samples. Both the redshift with the Ag coating and blueshift with the Al coating are large. These results differ from those of blue-emitting InGaN/GaN.

The green-emitting samples have a higher density of defects owing to the high indium composition, even in the polar samples, and the semi-polar samples have a higher percentage of defect density and dislocations caused by crystal growth. This higher defect density is an important factor in controlling the luminous properties of semi-polar InGaN with green emissions. Exciton localization should be the dominant factor in determining the emission properties of the semi-polar samples because the QCSE was canceled. In the green emission region, most of the localized excitons relax by non-radiative recombination because the indium-rich regions may contain many defects. As the exciton density increases, the non-radiative centers due to defects become saturated, and the emission becomes stronger. Therefore, understanding the effect of defects on the PL intensity and wavelength is necessary to understand the emission mechanism in semi-polar green-emission samples. Contrarily, the increase in the emission rate due to the Purcell effect, observed in the blue emission region, does not occur at the green emission wavelength. This absence is attributed to the SP wavelength at the Ag/GaN interface being too far from the green emission.

We considered the mechanism of the green emission from the semi-polar InGaN samples. In the metal-uncoated region, excitons are trapped at deep levels near the defects and are converted to heat by non-radiative recombination. In the case of the Ag-coated region, excitons in the deep levels near the defects can couple to the SP before being converted to heat and extracted as light emission. This is a possible mechanism for the redshift of the PL peak wavelength emitted from the deeper levels. The large wavelength shift obtained in the micro-PL measurement could be due to the large depth of the deep energy level at the defect site. Figure 6(d) shows the PL time profiles of the semi-polar sample with green emission of the InGaN/GaN QW. The PL lifetimes of the metal-uncoated, Ag-coated, and Al-coated regions were 0.32 ns, 0.43 ns, and 0.29 ns, respectively. The PL lifetime in the Ag-coated region increased, suggesting that the light emission occurred at deep levels. The internal quantum efficiency is expected to improve with this luminescence mechanism.

In the case of the Al-coated sample, the exciton density increased, so the defect levels were filled with more excitons than in the metal-uncoated region. The higher levels emitted light through the coupling of excitons and the SP of Al. This occurs because the SP of Al can be coupled with both the excitation light and emission. The wavelength shift was larger in the semi-polar green-emitting samples because of the depth of the defect, and not because of the fluctuation of the potential in the exciton localization effect. The PL time profiles of the green emission from semi-polar InGaN were shortened for the Al coating, as shown in Figure 6(d). The radiation rate increased because deep nonradiant sites were filled and emitted from higher sites. In addition, SP resonance at the Al interface can increase the emission rate. Owing to these effects, the PL lifetime of the green emission of Al-coated semi-polar InGaN/GaN shown in Figure 6(d) is the shortest. Both of these effects can contribute to a higher light-emission efficiency. A schematic of the mechanism of PL enhancement by the cancelation and saturation of the non-radiative recombination centers with SP resonance is shown in Figure S5. We believe that the difference in mechanism between Ag and Al is largely attributed to the difference in their SP wavelengths. In Ag, the exciton density decreases due to the coupling with the SP resonance wavelength affecting emissions. Conversely, in Al, the SP coupling leads to a strongly excited state, thereby increasing the exciton density. As the exciton density decreases, emission from excitons is further promoted, resulting in emission from deeper energy levels and a red-shift, as shown in Figure S5. Conversely, with increased exciton density, the deeper energy levels saturate, and emission from higher energy levels is observed, leading to a blue shift.

Finally, we discuss the correlation between PL peak intensity and wavelength. The correlation between the PL peak intensity and wavelength of blue emission for both the polar (Figure 2(c) and (d)) and semi-polar (Figure 4(c) and (d)) samples showed negative correlations for the metal-coated samples, and the correlation was almost flat for the uncoated samples. This may be because of the wavelength dependence of the SP resonance effect. As mentioned above, the SP resonance wavelengths at the Ag/GaN and Al/GaN interfaces were 440 and 250 nm, respectively. The PL enhancement effects of SP resonance become higher at wavelengths closer to these resonance wavelengths. Therefore, the negative correlation slopes were more remarkable for the Ag-coated sample in the blue emission region, which is closer to the SP resonance wavelength. Furthermore, for the green emissions far from the SP resonance wavelength, the negative correlations observed in the blue emissions were no longer observed for both the polar (Figure 7(c) and (d)) and semi-polar (Figure 9(c) and (d)) samples. This suggests that the SP resonance and emission wavelengths must be almost equal to obtain efficient SP enhancement effects, even if their detailed enhancement mechanisms are different. Therefore, the flexible tuning of the SP resonance wavelength is an important issue that should be addressed in future research.

To understand the enhancement of the IQE, temperature-dependent measurements of PL intensity are essential [38], [39], [40]. We have previously reported increases in both IQE and LEE for polar samples, enhanced with Ag and Al, based on temperature-dependent PL intensity measurements. IQE increases can also be estimated by determining the Purcell factor from the temperature-dependent emission lifetimes. Similar measurements are needed for semipolar samples, where preliminary findings indicate a unique temperature dependence, distinct from that of polar samples. A new model to explain this behavior quantitatively will be detailed in our upcoming report.

In recent years, InGaN/GaN LEDs have achieved high-efficiency emission in green [41], yellow-green [42], and even red light [43], drawing significant attention. The next major challenge is to develop and fabricate LEDs with practical, high efficiency using surface plasmon resonance, competitive with conventional methods.

## 4 Conclusions

In this study, we found that Ag and Al coatings enhanced the blue and green light emissions from polar and semi-polar InGaN/GaN QWs for each combination. Therefore, our proposed high-efficiency light emission by SP resonance is effective for semi-polar InGaN/GaN and is expected to be applied to high-efficiency LEDs. The SP resonance wavelength at the Ag/GaN interface is in the blue to green region, which resonates with the emission process and increases the emission rate. Furthermore, the SP resonance at the Al/GaN interface is in the ultraviolet region, which resonates with not only the emission but also the excitation light and enhances the density of the excitons generated. This affects the exciton localization effect, QCSE screening, and defect level saturation, indicating that SP resonance can control not only the emission efficiency, but also the exciton dynamics. In particular, green emission from the semi-polar InGaN/GaN QW is suitable for SP enhancement because of its high enhancement level and the possibility of reducing the effect of defects. We expect that SP resonance can be used to improve the efficiency of semi-polar LEDs in the future.

## Supplementary Material

See the supplementary material for the AFM images of deposited metal surfaces, the Gaussian fitting of the PL spectrum, and mechanisms of PL enhancements with QCSE, exciton localization, and SP resonance.

## Supplementary Material

Supplementary Material Details
