# Supplementary material for: Spatial and time-resolved properties of emission enhancement in polar/semi-polar InGaN/GaN by surface plasmon resonance
Source: Nanophotonics. 2024 Feb 7;13(8):1435–47. doi: 10.1515/nanoph-2023-0758 (PMC11635936; doi:10.1515/nanoph-2023-0758)
Supplement: Supplementary file 1 — Supplementary Material Details [file j_nanoph-2023-0758_suppl_001.pdf]

Supplementary Material

Spatial and Time-Resolved Properties of Emission Enhancement in Polar/Semi-Polar InGaN/GaN by Surface Plasmon Resonance

K. Ikeda[a], K. Kawai[a], J. Kametani[a], T. Matsuyama[a], K. Wada[a], N. Okada[b], K. Tadatomo[b], K. Okamoto[a]*

[a] Department of Physics and Electronics, Osaka Metropolitan University, Gakuen-cho, Naka-ku, Sakai-shi, Osaka 599-8531, Japan

[b] Department of Science and Technology for Innovation, Yamaguchi University, Tokiwadai, Ube-shi, Yamaguchi 755-8611, Japan

* *Corresponding author: OkamoT@omu.ac.jp*

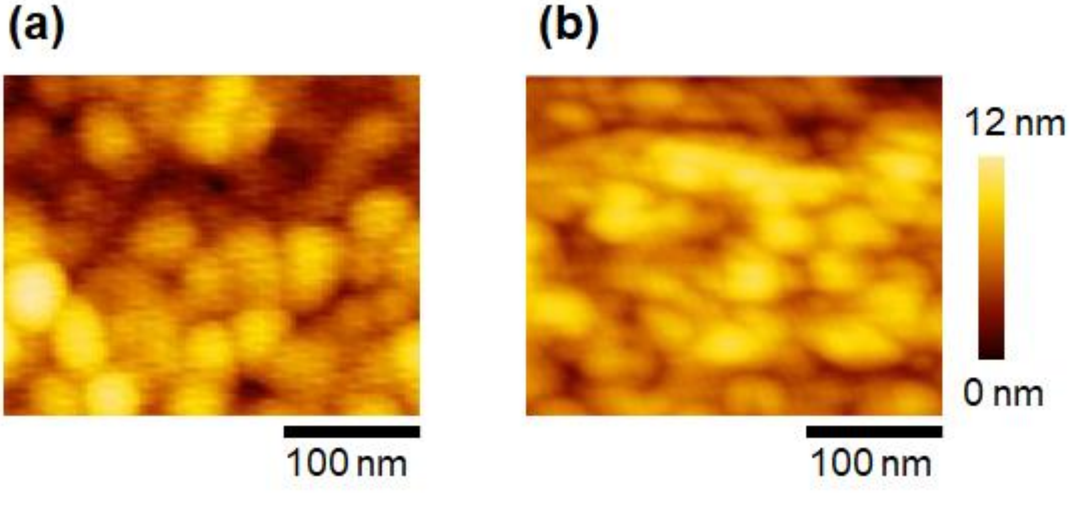

Fig. S1 AFM images of Ag (a) and Al (b) surfaces deposited on the samples using the high-vacuum thermal evaporation.

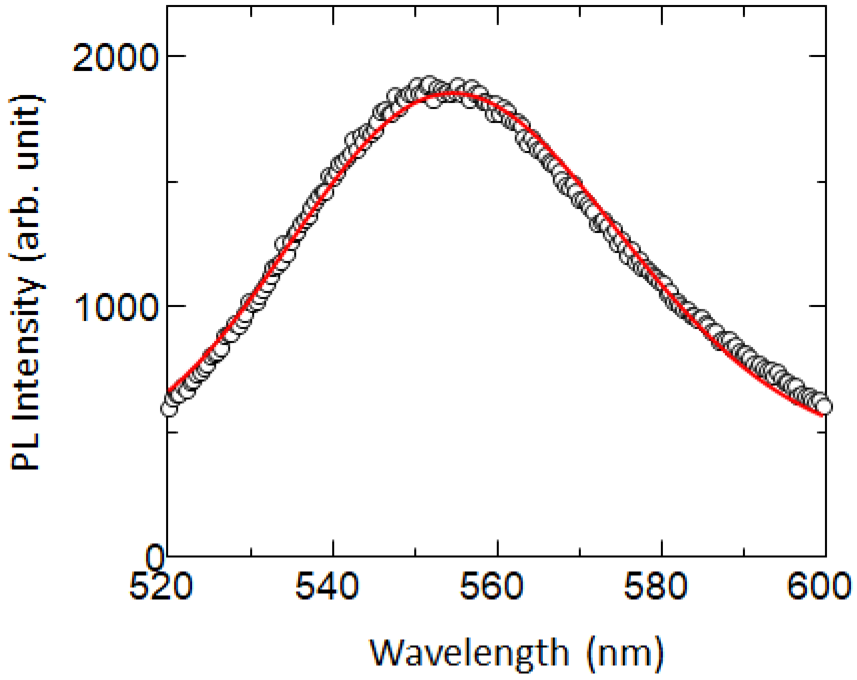

Fig. S2 Gaussian fitting of the typical PL spectrum in the wavelength regions near the PL peaks.

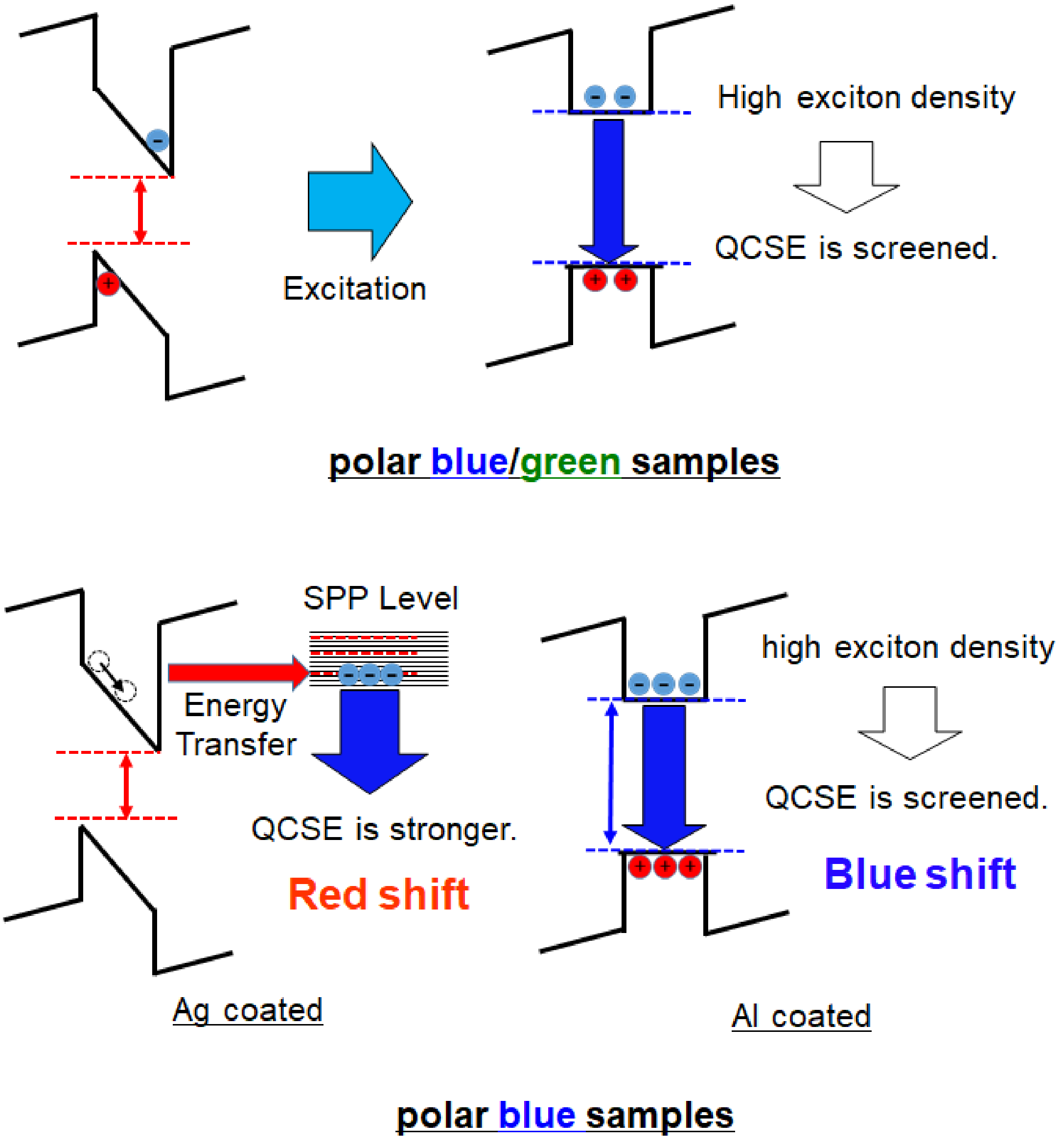

Fig. S3 Mechanism of PL enhancement by enhancement and reduction of the QCSE with surface plasmon resonance for polar blue/green samples. For the blue sample, the PL peaks are shifted by the SP resonance with Ag or Al coatings, but for the green sample, the QCSE is too strong to be much affected by SP resonance.

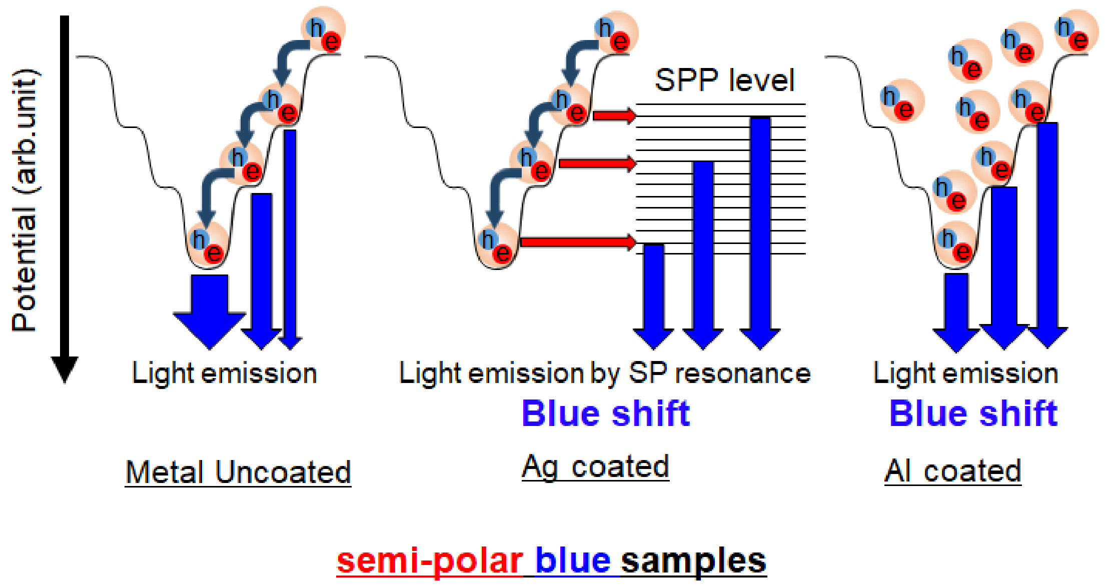

Fig. S4 Mechanism of PL enhancement by cancelation and saturation of the exciton localization effect with surface plasmon resonance for semi-polar blue samples.

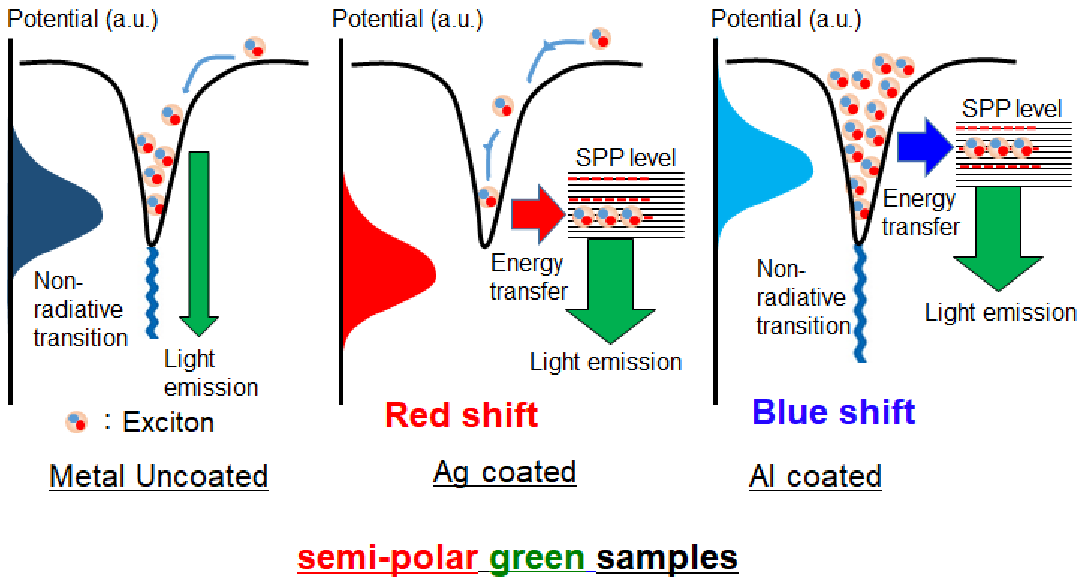

Fig. S5 Mechanism of PL enhancement by cancelation and saturation of the nonradiative recombination centers with surface plasmon resonance for semi-polar green samples.
